# Outcomes of Patients with Postoperative Acute Kidney Injury After Acute Type A Aortic Dissection Repair

**DOI:** 10.3390/jpm15010009

**Published:** 2024-12-28

**Authors:** George Samanidis, Kyriaki Kolovou, Meletios Kanakis, Sotirios Katsaridis, Konstantinos Perreas

**Affiliations:** 1 First Department of Adult Cardiac Surgery, Onassis Cardiac Surgery Center, 356 Leoforos Syggrou, 17674 Athens, Greece; katsaridisdoc@yahoo.gr (S.K.); perreas@doctors.org.uk (K.P.); 2 Department of Cardiac Surgery Intensive Care, Onassis Cardiac Surgery Center, 17674 Athens, Greece; sandykolovou@gmail.com; 3 Department of Pediatric and Congenital Heart Surgery, Onassis Cardiac Surgery Center, 17674 Athens, Greece; meletis_kanakis@yahoo.gr

**Keywords:** aortic dissection, thoracic aorta, acute kidney injury

## Abstract

**Introduction**: Acute type A aortic dissection (ATAAD) repair is associated with high morbidity postoperatively. The aim of this study is to evaluate the incidence and risk factors for acute kidney injury in patients who underwent ATAAD repair. **Patients and Methods**: Two hundred and twenty-three patients underwent ATAAD repair. Postoperative acute kidney injury (AKI) was evaluated according to the Kidney Disease—Improving Global Outcomes (KDIGO) criteria. **Results:** Postoperative AKI was observed in 140 patients (62.8%). The patients with postoperative AKI classified by KDIGO stages: 1 = 53 (23.8%), 2 = 36 (16.1%), and 3 = 51 (22.9%) patients. Twenty-eight patients (12.6%) underwent replacement renal therapy due to severe renal impairment (KDIGO stage 3). Multivariable logistic regression analysis (adjusted to risk factors) showed that preoperative eGFR was the risk factor for postoperative RRT (odds ratio (OR) = 0.95, 95% CI: 0.92–0.97, *p* < 0.01). The lengths of hospital and intensive care-unit stay differed between the patients with and without postoperative RRT (*p* < 0.001 for both). Postoperative RRT was associated with 30-day mortality (10.3% versus 35.7%, *p* < 0.001). **Conclusions**: Postoperative AKI was associated with high morbidity and mortality rate in patients after ATAAD repair.

## 1. Introduction

Acute type A aortic dissection (ATAAD) repair is associated with a wide spectrum of postoperative complications, as well as with high mortality rate. Postoperative neurological, renal, cardiac, respiratory, and hematologic dysfunctions affect the short-, mid-, and long-term outcomes of patients who undergo ATAAD repair. Prolonged length of intensive care (ICU) and hospital stay is a consequence of severe perioperative complications [1,2,3]. Postoperative acute kidney injury (AKI) in patients who are treated for ATAAD by intermittent hypothermic circulatory arrest (HCA) with or without cerebral perfusion contributes negatively to morbidity and mortality. Increase in postoperative serum creatinine, decrease in urine output, and replacement renal therapy (RRT) are present in 20–30% of patients with ATAAD. Furthermore, preoperative renal dysfunction is considered as a risk factor for presenting AKI postoperatively [4,5].

The incidence of postoperative acute kidney injury and the risk factors for new renal replacement therapy in patients who underwent ATAAD repair with hemiarch replacement under hypothermic circulatory arrest are discussed in this study.

## 2. Patients and Methods

### 2.1. Study Population

Between 2002 and 2022, two hundred and twenty-three patients underwent ATAAD repair with hemiarch replacement under moderate hypothermic circulatory arrest with antegrade cerebral perfusion (MHCA/ACP) or deep hypothermic arrest with retrograde cerebral perfusion (DHCA/RCP).

All perioperative data, including any postoperative renal dysfunction data, were recorded in our database. Inclusion criteria of the study were onset of symptoms < 14 days, age > 18 years old, and patients who underwent emergency operation < 24 h from admission to our hospital and patients who underwent hemiarch (proximal aortic arch) replacement only. Meanwhile, the exclusion criteria were patients who died in operating room (intraoperative), patients who underwent RRT (preoperative), and patients who were in cardiac arrest preoperatively. New postoperative AKI was evaluated according to Kidney Disease—Improving Global Outcomes (KDIGO) criteria [6]. In all patients, preoperative serum creatinine value and estimated glomerular filtration rate (eGFR), as well as the postoperative maximum serum creatinine and lower eGFR, were recorded. Preoperative and postoperative eGFR were calculated based on the Chronic Kidney Disease Epidemiology Collaboration (CKD-EPI) equation [7]. The patients with AKI on KDIGO stages 1 and 2 were treated conservatively with intravenous diuretic infusion, while the patients on KDIGO stage 3 who did not respond to conservative AKI treatment underwent invasive RRT (extracorporeal dialysis). Initiation and supervision of RRT were evaluated by specialist nephrologists. RRT was performed with a dual-lumen temporary hemodialysis catheter, which was placed in the right or left common femoral vein or left or right internal jugular vein. Two types of RRT were implemented: continuous venous–venous hemodialysis (CCHDF) and intermittent hemodialysis (IHD). Also, the number and type of RRT were recorded in our database.

Preoperative instability was defined when the mean systolic arterial pressure (MAP) <50 mmHg with persistent hypotension despite maximum vasoconstrictor and inotropic support.

### 2.2. Ethical Statement

This study was performed in accordance with the ethical standards of the Declaration of Helsinki, as revised in 2013. Also, the study was approved by the Ethics Committee or Institutional Review Board of Onassis Cardiac Surgery Center (546/30-04-2015). The need to receive informed consent from the patients was waived due to the nature of study (retrospective study).

### 2.3. Endpoints

Primary endpoints of study were the incidence of any AKI stages postoperative, incidence of postoperative RRT, risk factors for postoperative RRT, and short-term outcome of patients who underwent RRT, while secondary endpoints were intensive care unit (ICU) stay and length of stay in-hospital (LOS) of patients with or without AKI or RRT and 30-day mortality rate.

### 2.4. Surgical Techniques

Cardiopulmonary bypass was established with arterial cannulation (DHCA/RCP: common femoral artery (direct cannulation) or axillary artery (via synthetic graft 8 or 10 mm); MHCA/ACP: common carotid or axillary artery (via synthetic graft 8 or 10 mm) or femoral artery (direct cannulation)) and right atrium (two stage cannula) or bicaval cannulation. Cardiac arrest was achieved by retrograde infusion of cardioplegic solution.

In patients who underwent DHCA/RCP, the hemiarch or proximal aortic arch was repaired first without aortic cross clamp during circulatory arrest, and the proximal ascending aorta with/without aortic root repair and/or aortic valve replacement was performed afterwards with cross clamp. During DHCA, retrograde cerebral perfusion was achieved via catheter in the superior vena cava. During DHCA/RCP, the lowest bladder temperature was ≈18.7 °C.

On the other hand, in patients who underwent MHCA/ACP, the proximal ascending aorta with/without aortic root repair and/or aortic valve replacement was performed first with aortic cross clamp, and the proximal aortic arch or hemiarch was repaired afterwards under circulatory arrest. During MHCA, the antegrade cerebral perfusion was achieved via a synthetic graft in the common carotid artery (clamping proximal) and selective cannulation of the contralateral common carotid artery. During MHCA/ACP, the lowest bladder temperature was ≈22.5 °C.

### 2.5. Statistical Analysis

Distributed variables were expressed as median (Q1-Q3) and interquartile range (IQR) or mean ± standard deviation (SD). Qualitative variables were expressed as absolute and relative frequencies number (n) and (%). The normality of variable assumption was evaluated using Kolmogorov–Smirnov test. Student’s *t*-test, Mann–Whitney, and Kruskal–Wallis test were used for the comparison of continuous variables between groups of interest, and the type of test depended on the variable distribution (normal or not normal). For the comparisons of categorical variables, the proportions chi-square tests and Fisher’s exact tests were used. Wilcoxon signed-rank test was used to compare preoperative and postoperative eGFR. A 95% confidence interval (CI) was applied for all statistical tests. The possible risk factors for RRT postoperatively were evaluated by univariable and multivariable binary logistic regression analyses. Assessed risk factors for RRT that had *p* < 0.02 after univariable analysis were added to multivariable analysis. Statistical significance was set at *p* < 0.05, and IBM SPSS ver. 25 for Windows (IBM Corp., Armonk, NY, USA) was used for data analysis.

## 3. Results

### 3.1. Perioperative Details of Study Population

The median age of patients in cohort was 63 (53–71) years old. Females were 25% of study population. The median preoperative serum creatinine was 1 (0.8–1.4) mg/dL, while the median preoperative eGFR was 70 (49.5–87.5) mL/min/1.73 m^2^. Other preoperative characteristics of patients are presented in Table 1. Most of the patients, 136 (61%), underwent ATAAD correction under DHCA/RCP, and the most common arterial cannulation site was common femoral artery in 138 (61.9%) patients. Fifty-four patients (24.2%) transferred to the ICU with an open sternum due to severe hemorrhage, despite maximum coagulation support. Additional intraoperative details are shown in Table 2. During in-hospital postoperative course, the median postoperative serum creatinine was 1.6 (1.1–3.6) mg/dL, while the median postoperative eGFR was 40 (21.7–63.5) mL/min/1.73 m^2^. Furthermore, the postoperative eGFR was significantly lower to compare the preoperative eGFR, 40 (21.33–64) and 70 (49.7–82.6) mL/min/1.73 m^2^, respectively (*p* < 0.001). As seen from the above parameters, the ATAAD repair negatively affected the renal function in many patients. Also, as presented in Table 3, 140 (62.8%) patients had acute renal dysfunction based on the KDIGO criteria from stage 1 to 3. From the patients with postoperative AKI who were classified as having stage 3 according to the KDIGO criteria, 28 (12.6%) patients underwent new replacement renal therapy. Severe new postoperative neurological dysfunctions (PNDs) and coma were observed in 40 (18%) patients in the study population, while overall 30-day mortality was 13.5%. Other postoperative outcomes of all patients are shown in Table 3.

### 3.2. Association Characteristics of Patients and Perioperative Details with and Without Acute Kidney Injury

Eighty-three (63%) patients developed AKI after operation. The age and body surface area (BSA) did not differ in patients with and without AKI (*p* = 0.37 and *p* = 0.15, respectively), while sex (female) was associated with AKI (*p* = 0.01). Differences in patients with and without AKI are listed in Table 4. Type of HCA, arterial cannulation site, and cerebral perfusion did not affect the postoperative AKI, while transfer of patients to the ICU with open sternum was correlated with postoperative AKI (*p* < 0.001). Also, the length of postoperative stay in the ICU and hospital was longer in patients with AKI (Table 4). The 30-day mortality rate was higher in patients with AKI than in patients without AKI (17.1% versus 7.2%, *p* = 0.04).

### 3.3. Association Characteristics of Patients and Perioperative Details with Degree of Acute Kidney Injury

For the study purpose, the study population was divided into four groups based present or absent postoperative acute kidney injury. The KDIGO classification was used for patient stratification (stage 1 = 53 patients, stage 2 = 36 patients, and stage 3 = 51 patients). As shown in Table 5, the patients who was classified as having stage 3 by KDIGO had higher preoperative serum creatinine and lower eGFR compared with the other patients (*p* < 0.05); meanwhile, age, BSA, and sex were not associated with postoperative AKI. With regard to intraoperative details, transfer of the patients to the ICU with open sternum after operation and longer circulatory arrest time were associated with the heaviest acute renal dysfunction (*p* < 0.001 and *p* < 0.01, respectively). On the other hand, the type of surgical approach (DHCA/RCP versus MHCA/ACP), lower temperature during circulatory arrest, and arterial cannulation site (femoral artery, common carotid artery, and axillary artery) did not affect the postoperative AKI (*p* = 0.36, *p* = 0.32, *p* = 0.66, *p* = 0.21, and *p* = 0.19, respectively). As expected, the patients who were classified as having an advanced KDIGO stage had a longer ICU and in-hospital stay (*p* < 0.001). Also, the 30-day mortality was higher in patients with worst acute renal failure (*p* < 0.001). In stage 3, the 30-day mortality was 33.3%. Other details of patients based on KDIGO classification are listed in Table 5.

### 3.4. Association Characteristics of Patients and Perioperative Details with and Without Postoperative Replacement Renal Therapy

From 51 patients who were on stage 3 by KDIGO classification, 28 (12.6%) patients underwent replacement renal therapy (RRT) postoperatively. The comparison of perioperative details of patients who underwent or not to RRT postoperatively is listed in Table 6. Preoperative renal dysfunction and preoperative hemodynamic instability status were associated with risk for RRT (for both *p* < 0.001). Furthermore, longer circulatory arrest (CA) time and transfer to ICU with open sternum were associated with postoperative RRT. In addition, the lengths of in-hospital and ICU stay were longer in patients who underwent RRT than those who did not. Thirty-day mortality was higher in patients who required RRT (*p* = 0.001). Multivariable logistic regression analysis (adjusted to risk factors) showed that preoperative eGFR was the risk factor for postoperative RRT (odds ratio (OR) = 0.95, 95% CI: 0.92–0.97, *p* < 0.01) (Table 7).

### 3.5. Outcomes of Patients Who Underwent Postoperative Replacement Renal Therapy

Table 6 shows that the lengths of ICU and in-hospital stays were significantly different between the patients who underwent and those who did not undergo RRT postoperatively (*p* < 0.001 and *p* < 0.001, respectively). Thirty-day mortality rate was significantly different in the group without and the group with RRT (10.3% versus 35.7%, respectively) (*p* < 0.001). The short outcomes of patients who underwent RRT are presented in Table 8. Out of the 28 patients who underwent RRT, 10 (35.7%) patients died in-hospital within 30 days from the operation, 3 (10.7%) patients died in-hospital 30 days after operation, 6 (21.4%) patients were transferred to another hospital for rehabilitation, and 10 (35.7%) patients discharged at home. Although the number of patients who received any RRT was small, the 30-day mortality was lower in patients who received IHD (intermittent hemodialysis) than continuous veno-venous hemodialysis (CCVHDF) or both (CCVHDF plus IHD) (13.3%, 71.4% and 50%, respectively) (*p* = 0.02) (Table 8). Also, the patients who received IHD had more favorable short-term outcomes (discharge at home and transfer to another hospital for rehabilitation, 80% of patients) than CCVHDF or CCVHDF plus IHD. Regarding the fifteen patients who received only IHD in the group of patients with any RRT, the 30-day mortality rate was 13.3%, which is close to the 30-day mortality rate in patients without any RRT (including all patients with or without any AKI stages) (10.3%) (Table 6 and Table 8). Addition analysis shows that 30-day mortality did not differ between 15 patients who underwent IHD and the other 208 patients of study population, 2 patients (6.7%) and 13 patients (6.7%), respectively (*p* = 1.00).

## 4. Discussion

This study, like others, supports the high incidence of AKI after ATAAD surgery and highlights the impact of these serious complications on morbidity and mortality for these patients [4,5]. Our rate of AKI after ATAAD repair is higher (62.8%) than that of other studies, which reported a rate ranging from 40% to 55% but defined AKI according to the risk of renal dysfunction, injury to kidney, failure or loss kidney function, and end-stage kidney disease renal dysfunction (RIFLE) criteria [4,8]. However, there are studies that are in line with our AKI rate, although they defined AKI according to the Acute Kidney Injury Network (AKIN) criteria [9]. Increased rate of AKI was found in patients with preoperative renal dysfunction because of the dissection itself. Moreover, an increased risk of AKI was also found in patients who transferred to ICU with an open chest, and this can be explained by the higher number of blood transfusions. Both of these aforementioned risk factors are in accordance with previous studies [4,8,9].

Preoperatively, risk factors for the development of AKI are unmodifiable and common as hypovolemic shock, cardiac malperfusion, and renal artery involvement [9,10]. The existing literature has reported that most renal functions of patients with unilateral artery involvement can be improved with a better prognosis after ATAAD repair without further specific interventional treatment. In contrast, for cases involving bilateral renal arteries, postoperative renal function is often unsatisfactory; continued RRT or even further local renal intervention is required to improve renal vascular supply and, subsequently, renal function [11]. In this study, aortic dissection involving renal arteries as a risk factor in the final AKI risk prediction model was not included as a risk factor. Although AKI after ATAAD surgical repair is multifactorial and not necessarily directly related to preoperative renal malperfusion [11], our results derived from multivariable logistic regression analysis clearly support that preoperative eGFR was the risk factor for postoperative RRT. Our cohort patients with reduced preoperative kidney function were therefore at increased risk to develop the most severe form of AKI.

Open chest after surgical repair and, indirectly, the need for transfusion both seem to be markers of bleeding and complexity of surgery and well-known risk factors for AKI [12]. Transfusions have been shown to trigger proinflammatory states and increase oxidative stress and thereby increase the likelihood of AKI [13,14]. Hypothermia, cardiopulmonary bypass time, aortic cross clamp time, and age were not associated with AKI development in multivariable analysis.

AKI was associated with increased 30-day mortality, which is in concordance with a recent meta-analysis by Wang et al. [8]. There are scarce studies that describe less favorable long-term survival, but data on the long-term outcome of AKI patients after ATAAD surgical repair are lacking. Researchers have suggested that although renal function was relatively preserved, the patient with AKI had a longer hospital stay and required transfer for rehabilitation; subsequently, impaired activities of daily living may contribute to higher mortality in the long-term follow-up [15].

The 30-day mortality in ATAAD patients was 13.5%. The 30-day mortality rate in patients with AKI was 17.1% compared to 7.2% in patients without AKI. Although the mortality seems to increase with disease severity based on KDIGO criteria [5], in our study, the 30-day mortality for KDIGO stages 1, 2, and 3 was 11.3%, 2.8%, and 33.3%, respectively. This apparently low mortality for stage 2 can be explained by the fact that some patients went to stage 3 from the initial stage 2. Moreover, in this group of patients, aggressive RRT was initiated. Our study, like previous studies, documented that the development of AKI after ATAAD repair even in its mild form negatively affected the disease outcome, such as mortality and morbidity rate. Established guidelines for the prevention of postoperative AKI in patients who underwent ATAAD repair do not exist. Hemodynamic optimization by maintaining the fluid balance perioperatively and avoiding nephrotoxic drug administration, especially in the early postoperative period, is important. Moreover, close renal function monitoring by means of evaluation of urine output and renal biomarkers and prevention of progressive and uncontrolled metabolic acidosis by keeping optimal mean arterial pressure for the target organs should be included in the armamentarium for the early identification of renal dysfunction for this group of patients [16,17].

### Study Limitations

Our research had several limitations. It was a retrospective study, the number of patients was small, and it was conducted in a single center. The enrolled patients did not have an accurate description from the preoperative computed tomography (CT) regarding renal artery anatomy (possible unilateral or bilateral artery involvement) due to the fact that that most of CT scan was chest CT only and not full-body CT or CT angiography, particularly in past years. Also, many of CTs were performed in province with low-resolution images such as emergency CT scan, particularly in the past. In addition, a postoperative CT angiography was not included; thus, postoperative perfusion of the kidneys compared to preoperative was not measured. Possible other confounding risk factors for postoperative AKI, such us perioperative amount of blood and other products transfusion, time from diagnosis to treatment (operation), possible peripheral arteriopathy, perioperative medication (nephrotoxic drugs, diuretics, vasoconstrictive, and inotropic support), preoperative left ventricular ejection fraction (LVEF; data available in 87 (39%) of patients), and type of diabetes mellitus (insulin or non-insulin dependent diabetes mellitus; data available in 22 (9.8%) patients), were not included in this study due to missing data.

## 5. Conclusions

Acute type A aortic dissection repair is associated with high morbidity and mortality rate. Preoperative malperfusion syndrome in target organs (central nervous system, brain, liver, and kidney) negatively affects the short-term outcome. Preoperative renal dysfunction, circulatory arrest time, and transfer to ICU with open sternum are predisposing risk factors for postoperative acute renal failure and the need for postoperative RRT. Also, our analysis shows that patients who underwent RRT by IHD had more favorable outcomes than patients who received other types of RRT. Furthermore, the patients who classified in advantage stages of AKI (stage 3) had longer ICU and hospital stay.

## Figures and Tables

**Table 1:** Preoperative details of study population.

Variables	Total Number of Patients N = 223
Age, median (IQR), years old	63 (53–71)
Body surface area, median (IQR), m^2^	2.00 (1.80–2.16)
Sex, female, n (%)	56 (25.1)
Preoperative serum creatinine, median (IQR), mg/dL	1.0 (0.8–1.4)
Preoperative eGFR, median (IQR), mL/min/1.73 m^2^	70 (49.5–87.5)
Preoperative hemodynamic instability status, n (%)	56 (25.1)
Patients with aortic valve regurgitation (mild to moderate), n (%)	42 (18.8)
Preoperative D-Dimer, median (IQR), μg/mL	5839 (3418–10,000)
Preoperative NT-proBNP, median (IQR), pg/mL	305 (174–813)
Previous cardiac surgery, n (%)	12 (5.4)

IQR = interquartile range; n = number; eGFR = estimated glomerular filtration rate; NT-proBNP = N-terminal-pro brain natriuretic peptide.

**Table 2:** Intraoperative details of study population.

Variables	Total Number of Patients N = 223
Type of hypothermic circulatory arrest, n (%) *DHCA/RCP* *MHCA/ACP*	*136 (61)* *87 (39)*
Aortic valve replacement, n (%)	10 (4.5)
Modified Bentall operation, n (%)	26 (11.7)
Arterial cannulation site, n (%) *Common femoral artery* *Common carotid artery* *Axillary artery*	*138 (61.9)* *70 (31.4)* *15 (6.7)*
Intra-aortic balloon pump insertion, n (%)	4 (1.8)
Transfer in ICU with open sternum, n (%)	54 (24.2)
Cardiopulmonary bypass time, median (IQR), min	201 (182–229)
Aortic cross clamp time, median (IQR), min	100 (80–118)
Circulatory arrest time, median (IQR), min	34 (27–45)
Temperature, median (IQR), °C	20 (18.3–23.3)

IQR = interquartile range; n = number; DHCA/RCP = deep hypothermic circulatory arrest with retrograde cerebral perfusion; MHCA/ACP = moderate hypothermic circulatory arrest with antegrade cerebral perfusion; ICU = intensive care unit.

**Table 3:** Postoperative details of study population.

Variables	Total Number of Patients N = 223
Postoperative atrial fibrillation, n (%)	66 (29.6)
Postoperative permanent pacemaker insertion, n (%)	6 (2.7)
Pericardial effusion needs reoperation within 30 days after operation, n (%)	15 (6.7)
Postoperative maximum serum creatinine within 7 days, median (IQR), mg/dL	1.6 (1.1–3.0)
Postoperative minimum eGFR within 7 days, median (IQR), mL/min/1.73 m^2^	40 (21.7–63.5)
KDIGO stages, n (%) *1* *2* *3*	*53 (23.8)* *36 (16.1)* *51 (22.9)*
New postoperative replacement renal therapy, n (%)	28 (12.6)
New postoperative neurological complications, n (%) *Temporary neurological dysfunction* *Permanent neurological dysfunction* *Irreversible brain damage and coma*	*32 (14.3)* *24 (10.8)* *16 (7.2)*
Intensive care unit stay >48 h, n (%)	208 (93.3)
Intensive care unit stay, median (IQR), days	4 (3–9)
Length of in-hospital stay, median (IQR), days	10 (8–17)
30-day mortality, n (%)	30 (13.5)

IQR = interquartile range; n = number; eGFR = estimated glomerular filtration rate; KDIGO = Kidney Disease—Improving Global Outcomes.

**Table 4:** Characteristics and perioperative details of patients with and without postoperative acute kidney injury.

Variables	Acute Kidney Injury Total Number of Patient N = 223		*p*-Value
	No N = 83	Yes N = 140	
Age, median (IQR), years old	62 (53–68.5)	63 (53–72)	0.37
Body surface area, median (IQR), m^2^	1.95 (1.76–2.16)	2.00 (1.84–2.16)	0.15
Sex, female, n (%)	29 (34.9)	27 (19.3)	0.01 *
Preoperative serum creatinine, median (IQR), mg/dL	0.9 (0.8–1.3)	1.1 (0.9–1.5)	0.10
Preoperative eGFR, median (IQR), mL/min/1.73 m^2^	73 (55.1–87.2)	67 (46.5–87.8)	0.48
Preoperative hemodynamic instability status, n (%)	16 (19.3)	40 (28.6)	0.15
Previous cardiac surgery, n (%)	4 (4.8)	8 (5.7)	1.00
Type of hypothermic circulatory arrest, n (%) *DHCA/RCP* *MHCA/ACP*	48 (57.8) 35 (42.2)	88 (62.9) 52 (37.1)	0.48
Arterial cannulation sites *Femoral artery* *Axillary artery* *Carotid artery*	*53 (63.9)* *3 (3.6)* *27 (32.5)*	*85 (60.7)* *12 (8.6)* *43 (30.7)*	*0.67* *0.17* *0.88*
Aortic valve replacement, n (%)	6 (7.2)	4 (2.9)	0.18
Modified Bentall operation, n (%)	9 (10.8)	17 (12.1)	0.83
Intra-aortic balloon pump insertion, n (%)	0 (0)	4 (2.9)	0.29
Transfer in ICU with open sternum, n (%)	10 (12)	44 (31.4)	0.001 *
Cardiopulmonary bypass time, median (IQR), min	196 (177–229)	203 (185–230)	0.14
Aortic cross clamp time, median (IQR), min	93 (77.5–116)	100 (81.5–120)	0.26
Circulatory arrest time, median (IQR), min	35 (26–41)	34 (27–47.5)	0.46
Temperature, median (IQR), °C	20.5 (19–23.5)	20 (18–23.2)	0.30
Postoperative atrial fibrillation, n (%)	17 (20.5)	49 (35)	0.02 *
Postoperative permanent pacemaker insertion, n (%)	1 (1.2)	5 (3.6)	0.41
Postoperative maximum serum creatinine within 7 days, median (IQR), mg/dL	1.0 (0.8–1.2)	2.3 (1.6–4.4)	<0.001 *
Postoperative minimum eGFR within 7 days, median (IQR), mL/min/1.73 m^2^	66 (52.1–94.9)	28 (14–42)	<0.001 *
Intensive care unit stay >48 h, n (%)	77 (92.8)	131 (93.6)	0.79
Intensive care unit stay, median (IQR), days	3 (2–5.5)	5 (3–10)	<0.001 *
Length of in-hospital stay, median (IQR), days	9 (7–12)	12 (8–20.5)	0.001
30-day mortality, n (%)	6 (7.2)	24 (17.1)	0.04 *

IQR = interquartile range; N = number of patients; n = number; eGFR = estimated glomerular filtration rate; DHCA/RCP = deep hypothermic circulatory arrest with retrograde cerebral perfusion; MHCA/ACP = moderate hypothermic circulatory arrest with antegrade cerebral perfusion; ICU = intensive care unit; * = Statistical significance (*p* < 0.05).

**Table 5:** Characteristics of patients and perioperative details based on KDIGO classification.

Variables	KDIGO				*p*-Value
	No AKI N = 83	Stage 1 N = 53	Stage 2 N = 36	Stage 3 N = 51	
Age ± SD, years old	60 ± 13.7	59 ± 15.3	59.6 ± 12.1	65.3 ± 13	0.80
Body surface area ± SD, m^2^	1.94 ± 023	2.00 ± 0.23	2.02 ± 0.22	2.02 ± 0.28	0.17
Sex, female, n (%)	29 (34.9)	10 (18.9)	6 (16.7)	11 (21.9)	0.07
Preoperative serum creatinine ± SD, mg/dL	1.1 ± 0.8	1.1 ± 0.4	0.9 ± 0.2	1.4 ± 0.6	0.001 *
Preoperative eGFR ± SD, mL/min/1.73 m^2^	73.4 ± 29	74.7 ± 29	87.7 ± 36	57.8 ± 30	<0.001 *
Preoperative hemodynamic instability status, n (%)	17 (20.5)	12 (22.6)	8 (22.2)	19 (37.3)	0.15
Previous cardiac surgery, n (%)	4 (4.8)	3 (5.7)	0 (0)	5 (9.8)	0.25
Type of hypothermic circulatory arrest, n (%) *DHCA/RCP*	48 (57.8)	37 (69.8)	19 (52.8)	32 (62.7)	0.36
Arterial cannulation site, n (%) *Common femoral artery* *Common carotid artery* *Axillary artery*	53 (63.9) 27 (32.5) 3 (3.6)	33 (62.3) 13 (24.5) 6 (11.3)	19 (52.8) 16 (44.4) 1 (2.8)	33 (64.7) 14 (27.5) 5 (9.8)	0.66 0.21 0.19
Aortic valve replacement, n (%)	7 (8.4)	2 (3.8)	0 (0)	1 (2)	0.13
Modified Bentall operation, n (%)	9 (10.8)	10 (18.9)	3 (8.3)	4 (7.8)	0.28
Intra-aortic balloon pump insertion, n (%)	0 (0)	4 (7.5)	0 (0)	0 (0)	<0.01 *
Transfer in ICU with open sternum, n (%)	11 (13.3)	11 (20.8)	7 (19.4)	25 (49)	<0.001 *
Cardiopulmonary bypass time ± SD, min	203 ± 49	217 ± 67	211 ± 34	211 ± 47	0.49
Aortic cross clamp time ± SD, min	98 ± 41	99 ± 35	104 ± 31	109 ± 39	0.43
Circulatory arrest time, median (IQR), min	35 (26–41)	29 (24–38)	37 (29–45)	38 (31–54)	<0.01 *
Temperature ± SD, °C	21 ± 3.1	20 ± 3.2	21 ± 3.0	20 ± 3.1	0.32
Postoperative atrial fibrillation, n (%)	18 (21.7)	17 (32.1)	13 (36.1)	18 (35.3)	0.24
Postoperative maximum serum creatinine within 7 days ± SD, mg/dL	1.2 ± 1.0	1.7 ± 0.7	2.2 ± 0.8	5.4 ± 2.3	<0.001 *
Postoperative minimum eGFR within 7 days ± SD, mL/min/1.73 m^2^	71.7 ± 29	46.3 ± 17	34.6 ± 15	12.6 ± 6	<0.001 *
Intensive care unit stay >48 h, n (%)	77 (92.8)	45 (84.9)	35 (97.2)	51 (100)	0.01 *
Intensive care unit stay, median (IQR), days	3 (2–5)	4 (3–5)	4 (3–9)	13 (6–24)	<0.001 *
Length of in-hospital stay, median (IQR), days	9 (7–12)	9 (7–12)	11 (9–16)	18 (14–36)	<0.001 *
30-day mortality, n (%)	6 (7.2)	6 (11.3)	1 (2.8)	17 (33.3)	<0.001 *

IQR = interquartile range; N = number of patients; n = number; eGFR = estimated glomerular filtration rate; DHCA/RCP = deep hypothermic circulatory arrest with retrograde cerebral perfusion; MHCA/ACP = moderate hypothermic circulatory arrest with antegrade cerebral perfusion; ICU = intensive care unit; AKI = acute kidney injury; SD = standard deviation; KDIGO = Kidney Disease—Improving Global Outcomes; * = Statistical significance (*p* < 0.05).

**Table 6:** Characteristics and perioperative details of patients with and without postoperative replacement renal therapy (dialysis).

Variables	Replacement Renal Therapy (Dialysis) Total Number of Patient N = 223		*p*-Value
	No N = 195	Yes N = 28	
Age, median (IQR), years old	63 (52–70)	64.5 (57.5–77.5)	0.07
Body surface area, median (IQR), m^2^	2.00 (1.80–2.11)	1.98 (1.83–2.25)	0.37
Sex, female, n (%)	48 (24.6)	8 (28.6)	0.64
Preoperative serum creatinine, median (IQR), mg/dL	1 (0.8–1.3)	1.7 (1.3–1.9)	<0.001 *
Preoperative eGFR, median (IQR), mL/min/1.73 m^2^	74.3 (55.2–90)	40 (35.4–45.8)	<0.001 *
Preoperative hemodynamic instability status, n (%)	43 (22.1)	13 (46.4)	<0.001 *
Previous cardiac surgery, n (%)	9 (4.6)	3 (10.7)	0.18
Type of hypothermic circulatory arrest, n (%) *DHCA/RCP* *MHCA/ACP*	117 (60) 78 (40)	19 (67.9) 9 (32.1)	0.53
Aortic valve replacement, n (%)	9 (4.6)	1 (3.6)	1.00
Modified Bentall operation, n (%)	23 (11.8)	3 (10.7)	1.00
Intra-aortic balloon pump insertion, n (%)	4 (2.1)	0 (0)	1.00
Transfer in ICU with open sternum, n (%)	40 (20.5)	14 (50)	<0.01 *
Cardiopulmonary bypass time, median (IQR), min	200 (178–229)	207.5 (192–246)	0.09
Aortic cross clamp time, median (IQR), min	95 (78–116)	112 (83–136)	0.11
Circulatory arrest time, median (IQR), min	34 (26–42)	43 (31–59.5)	<0.01 *
Temperature, median (IQR), °C	20.5 (18.5–23.5)	19.4 (17.8–22.5)	0.19
Postoperative atrial fibrillation, n (%)	55 (29.1)	11 (32.4)	0.68
Postoperative permanent pacemaker insertion, n (%)	5 (2.6)	1 (2.9)	1.00
Postoperative maximum serum creatinine within 7 days, median (IQR), mg/dL	1.4 (1.0–2.1)	6.0 (4.4–7.5)	<0.001 *
Postoperative minimum eGFR within 7 days, median (IQR), mL/min/1.73 m^2^	49 (30.4–66.3)	9.05 (7.1–12.7)	<0.001 *
Intensive care unit stay >48 h, n (%)	180 (92.3)	28 (100)	0.12
Intensive care unit stay, median (IQR), days	4 (3–7)	19 (8–31)	<0.001 *
Length of in-hospital stay, median (IQR), days	10 (8–15)	24 (15–47)	<0.001 *
30-day mortality, n (%)	20 (10.3)	10 (35.7)	<0.001 *

IQR = interquartile range; N = number of patients; n = number; eGFR = estimated glomerular filtration rate; DHCA/RCP = deep hypothermic circulatory arrest with retrograde cerebral perfusion; MHCA/ACP = moderate hypothermic circulatory arrest with antegrade cerebral perfusion; ICU = intensive care unit; * = Statistical significance (*p* < 0.05).

**Table 7:** Multivariable logistic regression analysis of risk factors for postoperative invasive replacement renal therapy.

Variables	*p*-Value	OR	95% CI Lower and Upper Limits
Age (years old)	0.77	1.06	0.96–1.07
Preoperative eGFR (mL/min/1.73 m^2^)	<0.01 *	0.95	0.92–0.97
Preoperative hemodynamic instability	0.23	1.87	0.66–5.28
Type of hypothermic circulatory arrest	0.43	2.00	0.30–16.20
Femoral arterial cannulation	0.70	0.72	0.13–3.85
Carotid artery cannulation	0.46	0.47	0.05–3.62
Cardiopulmonary bypass time (min)	0.86	0.99	0.98–1.00
Aortic cross clamp time (min)	0.97	1.00	0.99–1.02
Circulatory arrest time (min)	0.06	1.02	0.99–1.04
Lowest temperature during circulatory arrest (°C)	0.57	0.93	0.72–1.19
Transfer to ICU with open sternum	0.10	2.25	0.83–6.06

DHCA/RCP = deep hypothermic circulatory arrest and retrograde cerebral perfusion; MHCA/ACP = moderate hypothermic circulatory arrest and antegrade cerebral perfusion; OR = odds ratio; CI = confidence interval; eGFR = estimated glomerular filtration rate; ICU = intensive care unit; * = Statistical significance (*p* < 0.05).

**Table 8:** Outcomes of patients who underwent replacement renal therapy.

Variables	CCVHDF N = 7	IHD N = 15	CCVHDF and IHD N = 6
Sessions, median (IQR), number	N/A	5 (1–37)	1 (1–10)
Overall hours, median (IQR), hours	165 (26.7–459)	N/A	165 (70–391)
Discharge at home, n (%)	1 (14.3)	6 (40)	2 (33.3)
Transfer to another hospital for rehabilitation, n (%)	0 (0)	6 (40)	0 (0)
30-day mortality, n (%)	5 (71.4)	2 (13.3)	3 (50)
In hospital mortality after 30-day, n (%)	1 (14.3)	1 (6.7)	1 (16.6)

CCHDF = continuous venous–venous hemodialysis; IHD = intermittent hemodialysis; IQR = interquartile range; N = number of patients; n = number; N/A = not applicable.

## Data Availability

The dataset of this study is available from the corresponding author upon reasonable request.

## References

[1] Matniyaz Y., Luo Y.X., Jiang Y., Zhang K.Y., Wang W.Z., Pan T., Wang D.J., Xue Y.X. (2024). Short- and Long-term survival prediction in patients with acute type A aortic dissection undergoing open surgery. J. Cardiothorac. Surg..

[2] Böning A., Kretzer J.A., Arif R., Etz C.D., Pöling J., Rylski B., Czerny M., Brickwedel J., Peterss S., Holubec T. (2024). Risk factors for long-term mortality after acute aortic dissection-results of the german registry for acute aortic dissection type a long-term follow-up. Eur. J. Cardiothorac. Surg..

[3] Park Y.K., Lee J.H., Kim K.M., Jung J.C., Chang H.W., Kim D.J., Kim J.S., Lim C., Park K.H. (2024). Acute type A aortic dissection features and outcomes in octogenarians: A propensity score analysis. Interdiscip. Cardiovasc. Thorac. Surg..

[4] Helgason D., Helgadottir S., Ahlsson A., Gunn J., Hjortdal V., Hansson E.C., Jeppsson A., Mennander A., Nozohoor S., Zindovic I. (2021). Acute Kidney Injury After Acute Repair of Type A Aortic Dissection. Ann. Thorac. Surg..

[5] Wang Z., Ge M., Chen T., Chen C., Zong Q., Lu L., Wang D. (2020). Acute kidney injury in patients operated on for type A acute aortic dissection: Incidence, risk factors and short-term outcomes. Interact. Cardiovasc. Thorac. Surg..

[6] Khwaja A. (2012). KDIGO clinical practice guidelines for acute kidney injury. Nephron Clin. Pract..

[7] Inker L.A., Eneanya N.D., Coresh J., Tighiouart H., Wang D., Sang Y., Crews D.C., Doria A., Estrella M.M., Froissart M. (2021). New Creatinine- and Cystatin C-Based Equations to Estimate GFR without Race. N. Engl. J. Med..

[8] Wang Y., Yu L., Wang T. (2018). *MicroRNA*-374b inhibits the tumor growth and promotes apoptosis in non-small cell lung cancer tissue through the p38/ERK signaling pathway by targeting JAM-2. J. Thorac. Dis..

[9] Zhao H., Pan X., Gong Z., Zheng J., Liu Y., Zhu J., Sun L. (2015). Risk factors for acute kidney injury in overweight patients with acute type A aortic dissection: A retrospective study. J. Thorac. Dis..

[10] Zhang Y., Lan Y., Chen T., Chen Q., Guo Z., Jiang N. (2022). Prediction of Acute Kidney Injury for Acute Type A Aortic Dissection Patients Who Underwent Sun’s Procedure by a Perioperative Nomogram. Cardiorenal Med..

[11] Czerny M., Schoenhoff F., Etz C., Englberger L., Khaladj N., Zierer A., Weigang E., Hoffmann I., Blettner M., Carrel T.P. (2015). The Impact of Pre-Operative Malperfusion on Outcome in Acute Type A Aortic Dissection: Results From the GERAADA Registry. J. Am. Coll. Cardiol..

[12] Stone G.W., Clayton T.C., Mehran R., Dangas G., Parise H., Fahy M., Pocock S.J. (2012). Impact of major bleeding and blood transfusions after cardiac surgery: Analysis from the Acute Catheterization and Urgent Intervention Triage strategY (ACUITY) trial. Am. Heart J..

[13] Karkouti K., Wijeysundera D.N., Yau T.M., Callum J.L., Cheng D.C., Crowther M., Dupuis J.Y., Fremes S.E., Kent B., Laflamme C. (2009). Acute kidney injury after cardiac surgery: Focus on modifiable risk factors. Circulation.

[14] Tinmouth A., Fergusson D., Yee I.C., Hébert P.C., ABLE Investigators, Canadian Critical Care Trials Group (2006). Clinical consequences of red cell storage in the critically ill. Transfusion.

[15] Kato A., Ito E., Kamegai N., Mizutani M., Shimogushi H., Tanaka A., Shinjo H., Otsuka Y., Inaguma D., Takeda A. (2016). Risk factors for acute kidney injury after initial acute aortic dissection and their effect on long-term mortality. Ren. Replace. Ther..

[16] Wang Z., Ge M., Chen T., Chen C., Zong Q., Lu L., Li K., Wang D. (2020). Risk factors and long-term outcomes of elderly patients complicating with acute kidney injury after type A acute aortic dissection surgery: A retrospective study. J. Thorac. Dis..

[17] Shin S.R., Kim W.H., Kim D.J., Shin I.W., Sohn J.T. (2016). Prediction and Prevention of Acute Kidney Injury after Cardiac Surgery. Biomed. Res. Int..

